# *CellPie*: a scalable spatial transcriptomics factor discovery method via joint non-negative matrix factorization

**DOI:** 10.1093/nar/gkaf251

**Published:** 2025-04-01

**Authors:** Sokratia Georgaka, William Geraint Morgans, Qian Zhao, Diego Sanchez Martinez, Amin Ali, Mohamed Ghafoor, Syed-Murtuza Baker, Robert G Bristow, Mudassar Iqbal, Magnus Rattray

**Affiliations:** Division of Informatics, Imaging and Data Sciences, Faculty of Biology, Medicine and Health, University of Manchester, Manchester M13 9PL, United Kingdom; Division of Informatics, Imaging and Data Sciences, Faculty of Biology, Medicine and Health, University of Manchester, Manchester M13 9PL, United Kingdom; Division of Informatics, Imaging and Data Sciences, Faculty of Biology, Medicine and Health, University of Manchester, Manchester M13 9PL, United Kingdom; CRUK Manchester Institute, University of Manchester, Manchester M20 4BX, United Kingdom; Division of Informatics, Imaging and Data Sciences, Faculty of Biology, Medicine and Health, University of Manchester, Manchester M13 9PL, United Kingdom; The Christie NHS Foundation Trust, Manchester M20 4BX, United Kingdom; Division of Informatics, Imaging and Data Sciences, Faculty of Biology, Medicine and Health, University of Manchester, Manchester M13 9PL, United Kingdom; Division of Informatics, Imaging and Data Sciences, Faculty of Biology, Medicine and Health, University of Manchester, Manchester M13 9PL, United Kingdom; Division of Informatics, Imaging and Data Sciences, Faculty of Biology, Medicine and Health, University of Manchester, Manchester M13 9PL, United Kingdom; Division of Cancer Sciences, Faculty of Biology, Medicine and Health, University of Manchester, Manchester M13 9PL, United Kingdom; Division of Informatics, Imaging and Data Sciences, Faculty of Biology, Medicine and Health, University of Manchester, Manchester M13 9PL, United Kingdom; Division of Informatics, Imaging and Data Sciences, Faculty of Biology, Medicine and Health, University of Manchester, Manchester M13 9PL, United Kingdom

## Abstract

Spatially resolved transcriptomics has enabled the study of expression of genes within tissues while retaining their spatial identity. Most spatial transcriptomics (ST) technologies generate a matched histopathological image as part of the standard pipeline, providing morphological information that can complement the transcriptomics data. Here, we present *CellPie*, a fast, unsupervised factor discovery method based on joint non-negative matrix factorization of spatial RNA transcripts and histological image features. *CellPie* employs the accelerated hierarchical least squares method to significantly reduce the computational time, enabling efficient application to high-dimensional ST datasets. We assessed *CellPie* on three different human cancer types with different spatial resolutions, including a highly resolved Visium HD dataset, demonstrating both good performance and high computational efficiency compared to existing methods.

## Graphical Abstract

**Graphical Abstract:**
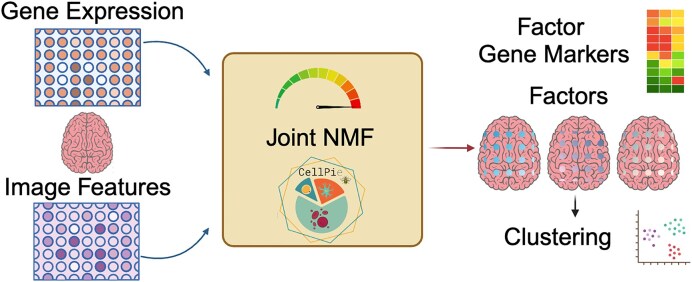

## Introduction

In multicellular organisms, tissues are complex systems composed of millions of cells that constitute the building blocks of whole organs. Within tissues, cells vary in type and activity, and their development and function are influenced by interactions with their surroundings. Therefore, dissecting spatial cellular organization and heterogeneity within tissues is important for understanding normal tissue function as well as diseases, which often have spatial origins [1].

Cutting-edge technologies, such as single-cell RNA sequencing (scRNA-seq), achieve high-throughput and high-resolution gene expression profiles, providing powerful insights into the characterization of the heterogeneity at the transcriptomic level [2, 3]. However, these methods dissociate the tissue, resulting in the loss of spatial dimension. *In situ* capture methods, such as spatial transcriptomics (ST) [4], commercially available as Visium by 10x Genomics, Slide-seq [5] (Slide-seqV2), and high-definition ST [6] (HDST), allow molecular profiling while retaining the spatial information of the tissue, at various resolutions [7, 8]. Slide-seq and HDST use beads (‘pucks’) to provide near-single-cell spatial resolution of 10 and 2 μm, respectively, while the Visium barcoded spots have a coarser resolution of 55 μm. Recently, 10x Genomics released the high-resolution Visium HD spatial gene expression platform [9], offering whole transcriptome spatial mapping at single-cell/near-single-cell resolution (available at 2, 8, and 16 μm).

ST data offer a multimodal view of the tissue landscape. *In situ* capture methods provide spatial and transcriptomic information along with matched haematoxylin and eosin (H&E) stained images as part of their standard pipelines. These histopathological images provide a comprehensive view of the tissue architecture that can complement spatial gene expression. Most current dimensionality reduction and factor analysis (FA) methods either model the gene expression modality alone or integrate together the spatial and gene expression parts, without utilizing the image modality. For example, dimensionality reduction methods, such as nonnegative spatial factorization (NSF) (and the NSF hybrid, NSFH) [10] and MEFISTO [11], integrate spatial and gene expression dimensions, while unimodal methods, such as probabilistic non-negative matrix factorization (PNMF) and FA [12], model gene expression alone. NSF is based on the non-negative spatial factorization model, which assigns an exponentiated Gaussian process (GP) prior over the spatial locations with either a Poisson or a negative binomial likelihood over the gene expression counts. MEFISTO performs a spatially aware FA through factorizing the gene expression data into latent GPs. While these methods scale reasonably well for Visium 10x datasets, when it comes to higher resolved ST datasets, such as the Visium HD, they become computationally prohibitive.

To address these limitations, we present *CellPie*, a flexible and scalable factor discovery tool based on unsupervised joint NMF (jNMF). Both joint and single NMF-based methods have become popular in single-cell genomics for producing interpretable sparse features from high-dimensional data. jNMF has previously been applied to integrate multiple transcriptomics datasets [13], omics profiles from observational (TCGA) and experimental (CCLE) data [14], and biomarker discovery [15]. *CellPie* jointly models spatial gene expression data and matched morphological imaging features, outputting a joint, parts-based representation (factors) of the high-dimensional data in a simple and computationally efficient way. We benchmarked *CellPie* against published dimensionality reduction methods on two human cancer ST datasets, a Visium 10x Genomics human prostate adenocarcinoma with invasive carcinoma and an ST HER2 (human epidermal growth factor receptor 2)-positive human breast cancer sample. Additionally, we applied *CellPie* to a Visium HD human colorectal cancer (CRC) sample, demonstrating its high computational performance, against standard NMF implementations [16].

## Materials and methods

### Joint non-negative matrix factorization

*CellPie* employs a scalable NMF-based approach, which offers a joint, parts-based representation of multiple modalities. Our method builds on the efficient unimodal NMF scheme proposed in [17], which solves the NMF problem using the accelerated hierarchical alternating least squares (A-HALS) algorithm. This algorithm has recently been modified to solve the jNMF problem (Equation 1, intNMF [18]). Here, we adapt the method to jointly factorize spatial gene expression and morphological image features derived from a matched H&E image. The joint factorization problem for spot-wise spatial gene expression and paired spot-wise morphological image feature data is formulated as follows.

Given two non-negative input matrices $Y_{\mathrm{rna}}\in \mathbb {R}{_{+}}^{m\times n}$ and $Y_{\mathrm{img}}\in \mathbb {R}{_{+}}^{m\times f}$, where *m* is the number of spatial locations (spots), *n* is the number of genes, and *f* the number of image features, building on the intNMF formulation, *CellPie* factorizes the high-dimensional input matrices into a shared non-negative matrix $W\in \mathbb {R}{_{+}}^{m\times k}$ and two modality-specific non-negative matrices $H_{\mathrm{rna}}\in \mathbb {R}{_{+}}^{k\times n}$, where *k* ≪ *n*, *f* is the number of factors (rank), and $H_{\mathrm{img}}\in \mathbb {R}{_{+}}^{k\times f}$ matrices, such that


(1) \begin{eqnarray*}
Y_{\mathrm{rna}}\approx WH_{\mathrm{rna}}, \nonumber \\ Y_{\mathrm{img}}\approx WH_{\mathrm{img}},
\end{eqnarray*}

for gene expression counts and image feature data, respectively.

The factors *W*, *H*_rna_, and *H*_img_ are calculated by solving the following joint optimization problem:


\begin{eqnarray*}
\min _{W,H_{\mathrm{rna}},H_{\mathrm{img}}} \alpha \Vert Y_{\mathrm{rna}} - WH_{\mathrm{rna}}\Vert _{\rm F}^2 + (2-\alpha ) \Vert Y_{\mathrm{img}} - WH_{\mathrm{img}} \Vert _{\rm F}^2 \ , \nonumber
\end{eqnarray*}

(2) \begin{eqnarray*}
\textrm {s.t.} \ W,H_{\mathrm{rna}},H_{\mathrm{img}}\ge 0 \ ,
\end{eqnarray*}

where the parameter *α* is a modality-specific weight and ‖·‖_F_ is the Frobenius norm. The modality-specific weight is by default set to 1.0 so that each modality is equally weighted. However, if a ground truth is available, for example pathologist’s annotated clusters, this parameter can be optimized, as we will discuss in the ‘Results’ section. If ground truth is not available, we encourage the user to run *CellPie* for a few different modality weights with values close to 1.0 (e.g. 0.8, 1.0 and 1.2) and select the weight that leads to the optimal desirable outcome.

The above non-convex optimization problem is reduced to an alternating trio of convex optimizations by fixing the matrices not being updated (e.g. solve for *W* fix *H*_rna_, *H*_img_). For details of the update algorithm (A-HALS modified to jointly factorize two modalities) we refer the reader to [18].

The image-based morphological features (*Y*_img_) are extracted from square patches centred at the spot locations of the corresponding H&E image. To capture morphological details across different spatial scales, features are extracted from a range of different patch sizes. For example, for Visium 10x datasets, where there is a gap of 45 μm between two neighbouring spots where spatial gene expression is not measured, the default patch size is set to a range between 0.1 and 3.0 of the spot diameter in order to include morphological information that compensates for the discontinuity of the gene expression between the spots. For datasets with coarser resolution, such as the ST, where the gap between two adjacent spots is larger, a broader range of patch sizes can be employed to capture more context around the spot. By default, *CellPie* uses spot-level pixel intensity features (bin-counts) of each colour channel in the H&E image. These are computed using histogram-based features derived from pixel intensity values within each patch. Specifically, Squidpy’s [19] extract histogram features function is used, which divides the intensity range of each channel into a fixed number of bins, counting the number of pixels within each bin interval and creating a binned intensity profile for each patch and colour channel. This summarizes spot-level intensity patterns and morphological details within each patch, allowing for the application of factorization algorithms.

**Figure 1.**
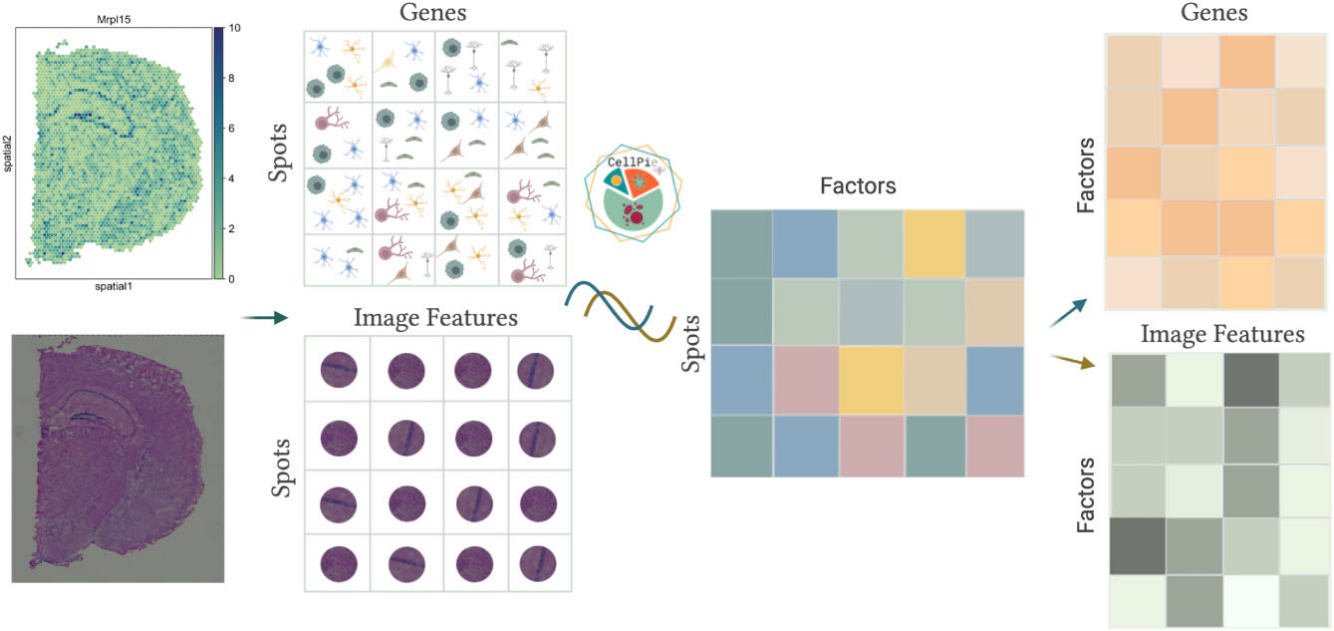
Graphical overview of the *CellPie* method. *CellPie* takes as input spatial gene expression counts (spots by genes) and paired morphological image features extracted from H&E images (spots by features). These two modalities are jointly factorized using *CellPie* , resulting in three matrices: a shared spots by factors matrix, containing the reduced parts-based representation (factors), and two individual matrices containing the weights of each of the features, a gene loading and an image loading matrix.

In addition to the default histogram-based morphological features, *CellPie* supports user-defined, non-negative, and spot-wise image features. These could include features extracted using deep learning techniques, such as convolutional neural networks. An overview of the *CellPie* approach is shown in Fig. 1.

### Model selection and initialization

To help the user select the optimal number of factors, *k*, *CellPie* implements model selection through the bi-cross-validation (BCV) method proposed in [20]. In this method, the gene expression matrix (*Y*_rna_) is partitioned into four random blocks, as follows:


\begin{eqnarray*}
Y_{\mathrm{rna}}={\begin{pmatrix}A & B \\ C & D \end{pmatrix}} ,
\end{eqnarray*}

where $A\in \mathbb {R}{_{+}}^{r\times s}$, $B\in \mathbb {R}{_{+}}^{r\times (n-s)}$, $C\in \mathbb {R}{_{+}}^{(m-r)\times s}$, and $D\in \mathbb {R}{_{+}}^{(m-r)\times (n-s)}$. Blocks *B*, *C*, and *D* are then used for training, while the upper-left block *A* is predicted from *D* using the off-diagonal *B* and *C* blocks. We run *CellPie* for a range of *k* values and the optimal model is considered the one with the lowest reconstruction error. However, it is worth pointing out that prior biological knowledge of the tissue is always advantageous, and *k* can also be selected in conjunction with this prior knowledge where available.

The intNMF algorithm is iterative and may converge to different solutions depending on the initialization of the matrices. By default, *CellPie* employs random initialization, where the matrix elements are sampled from a half-normal distribution (to ensure positivity). This choice ensures flexibility and adaptability across a diverse range of datasets. Random initialization is useful as it captures the natural variability inherent in biological data, allowing for unbiased exploration of the solution space.

As an alternative, *CellPie* also provides the option to use the non-negative double singular value decomposition (NNDSVD), a robust, non-random low-rank initialization strategy, proposed by Boutsidis and Gallopoulos [21]. This algorithm uses two SVD processes and has been demonstrated to rapidly reduce the approximation error. Despite these advantages, random initialization is preferable for this application due to its ability to avoid potential bias introduced by the constrained structures that are discovered by NNDSVD.

### Benchmarking and applications

We benchmarked *CellPie* on data with different spatial resolutions from two different human cancer types. In the first instance, we used a publicly available Visium human prostate adenocarcinoma with invasive carcinoma in which pathologist’s annotations are provided. The second validation considers a published ST dataset of a HER2-positive human breast cancer [22], where pathologist’s annotations of the tissue are also available. We evaluated the clustering performance of *CellPie* and other published dimensionality reduction methods (NSF, NSFH, MEFISTO, PNMF, and FA) against the pathologist’s ground truth labels. In addition, to demonstrate the scalability of the algorithm for very high dimensional datasets, we applied *CellPie* on a recent highly resolved Visium HD human CRC dataset [9]. More information on parameter settings for the competing methods is available in the supplementary material.

## Results

### Visium human prostate invasive carcinoma

**Figure 2.**
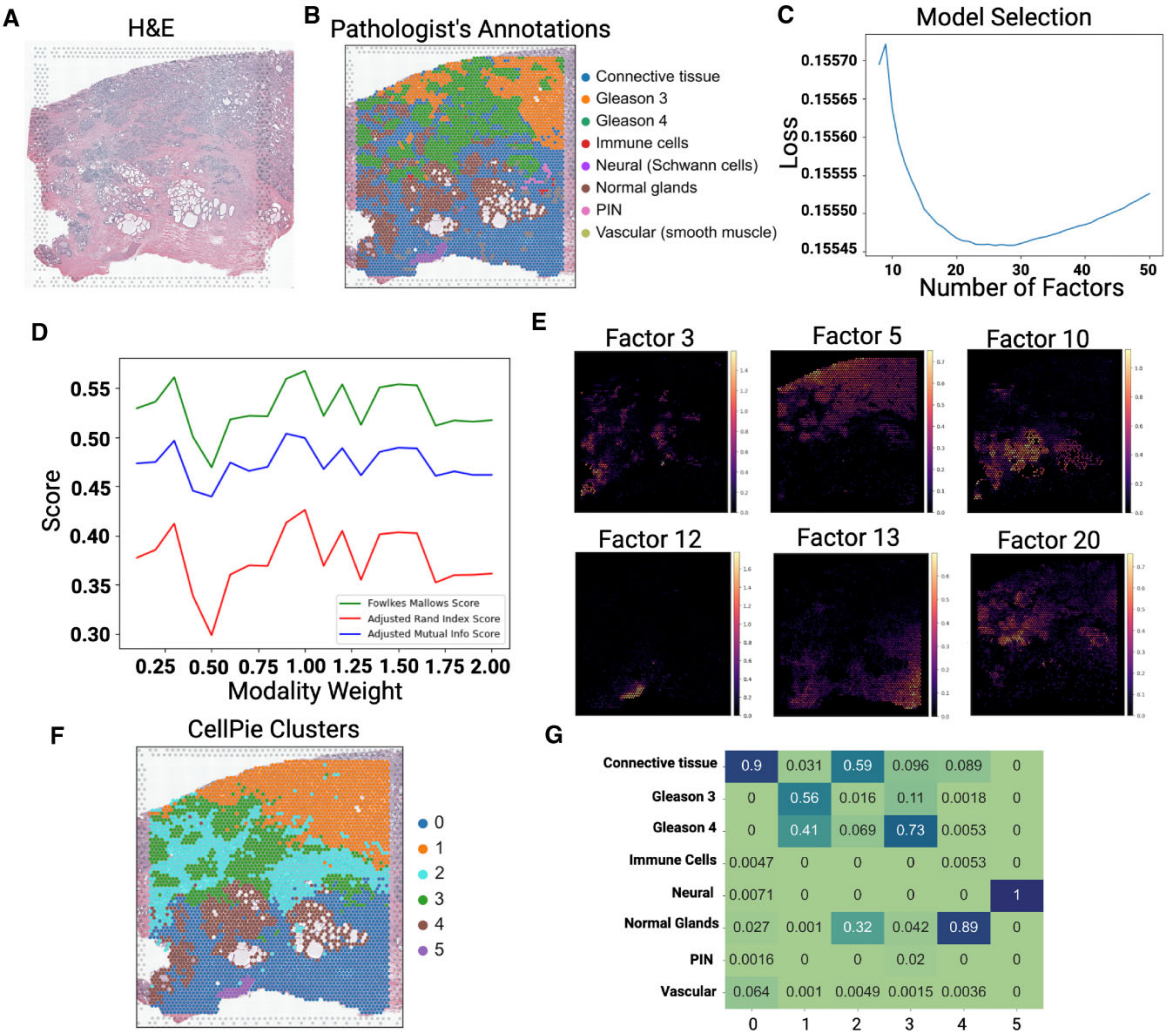
Validation of *CellPie* on human prostate cancer data. (**A**) H&E image of the invasive prostate carcinoma tissue. (**B**) Pathologist’s annotations of the tissue. (**C**) Model selection for *CellPie* for a range of factors. (**D**) Clustering performance of *CellPie* for a range of modality weights. The plot shows three measures [Fowlkes–Mallows, adjusted Rand index (ARI), and adjusted mutual Info] of clustering similarity between *CellPie* and the pathologist’s annotations. (**E**) *CellPie*’s selected factors that represent the pathologist’s annotated regions. (**F**) *CellPie* clusters computed using the Leiden algorithm on *CellPie*’s output factors. (**G**) Contingency table between *CellPie*’s clusters and pathologist’s annotations.

Initially, we applied and benchmarked *CellPie* on a 10x Genomics Visium human prostate adenocarcinoma with invasive carcinoma dataset (Fig. 2A). 10x Genomics supplies a paired H&E image that has been annotated by a pathologist. To achieve finer granularity of the invasive carcinoma region, we re-annotated the image with more fine-grained pathologist annotations of the different tissue regions (connective tissue, Gleason 3, Gleason 4, immune cells, neural, normal glands, PIN, and vascular) as shown in Fig. 2B. Consequently, the tumour region was annotated using the Gleason scoring system, labelled as Gleason 3 and Gleason 4. This enables us to evaluate whether *CellPie* can identify factors that correspond to these specific Gleason areas. In prostate, two main lineages that are biologically and architecturally different can be seen: the stromal compartment and the epithelial compartment. The stroma is composed of fibroblasts, smooth muscle, nerves, and blood vessels in diverse proportions [23, 24]. Prostate carcinoma arises from the glandular epithelial compartment and, unlike other organs, is not graded by individual cell differentiation but mainly by its architectural features using the Gleason grading, from 1 to 5. Due to poor reproducibility and lack of biological support, Gleason 1 and 2 are no longer reported. Gleason 3 cancers comprise the most differentiated adenocarcinoma, consisting of discrete glandular units with varying sizes and shapes [25]. Individual tumour acini have smooth, typically circular edges and intact basement membranes. In contrast, Gleason 4 cancers are composed of poorly formed glandular units with indistinct borders, fused glands, and irregularly infiltrating stroma [25, 26]. Intuitively, Gleason 4 tumours exhibit higher degrees of dedifferentiation, increased cancer progression, and therefore stronger prognostic correlations.

*CellPie* contains two hyper-parameters, the number of factors and the modality weight, *k* and *α*, respectively. To find the optimal number of factors, we used *CellPie*’s tailored model selection (BCV), which found that the optimal number is *k* = 26 (Fig. 2C). The optimal modality weight was at *α* = 1.0 (Fig. 2D). To find this, we clustered *CellPie*’s factors using the Leiden clustering algorithm, where we adjusted the number of neighbours and the resolution parameters to end up with six clusters corresponding to the six major pathologist’s annotated areas (PIN, immune, and vascular are only found in a few spots). We then investigated the optimal modality weight by calculating the ARI between the factor clusters and the pathologist’s labels (Fig. 2D). The results indicate that the integration of the two modalities leads to a better clustering performance than using a single modality. With these settings, selected *CellPie*’s factors that correspond to the pathologist’s annotations are shown in Fig. 2E (Supplementary Fig. S1 for all factors). We then clustered *CellPie*’s factors using Leiden clustering and the resulting clusters are shown in Fig. 2F.

To label the clusters, we computed the cross-tabulation between *CellPie*’s clusters and the pathologist’s annotations, where cluster 0 mainly corresponds to the connective tissue region and vascular region (Fig. 2G). In cluster 1, Gleason 3 predominates, interspersed with Gleason 4. Conversely, in cluster 3, Gleason 4 predominates, interspersed with a majority of PIN and a minority of Gleason 3. Cluster 4 aligns with the normal glands and cluster 5 corresponds to the neural region (Fig. 2G). Overall, *CellPie* can identify the six major tissue areas with a corresponding ARI of ≈0.43, showing good agreement with the ground truth (the fact that the connective tissue is split into two clusters results in a lower ARI).

**Figure 3.**
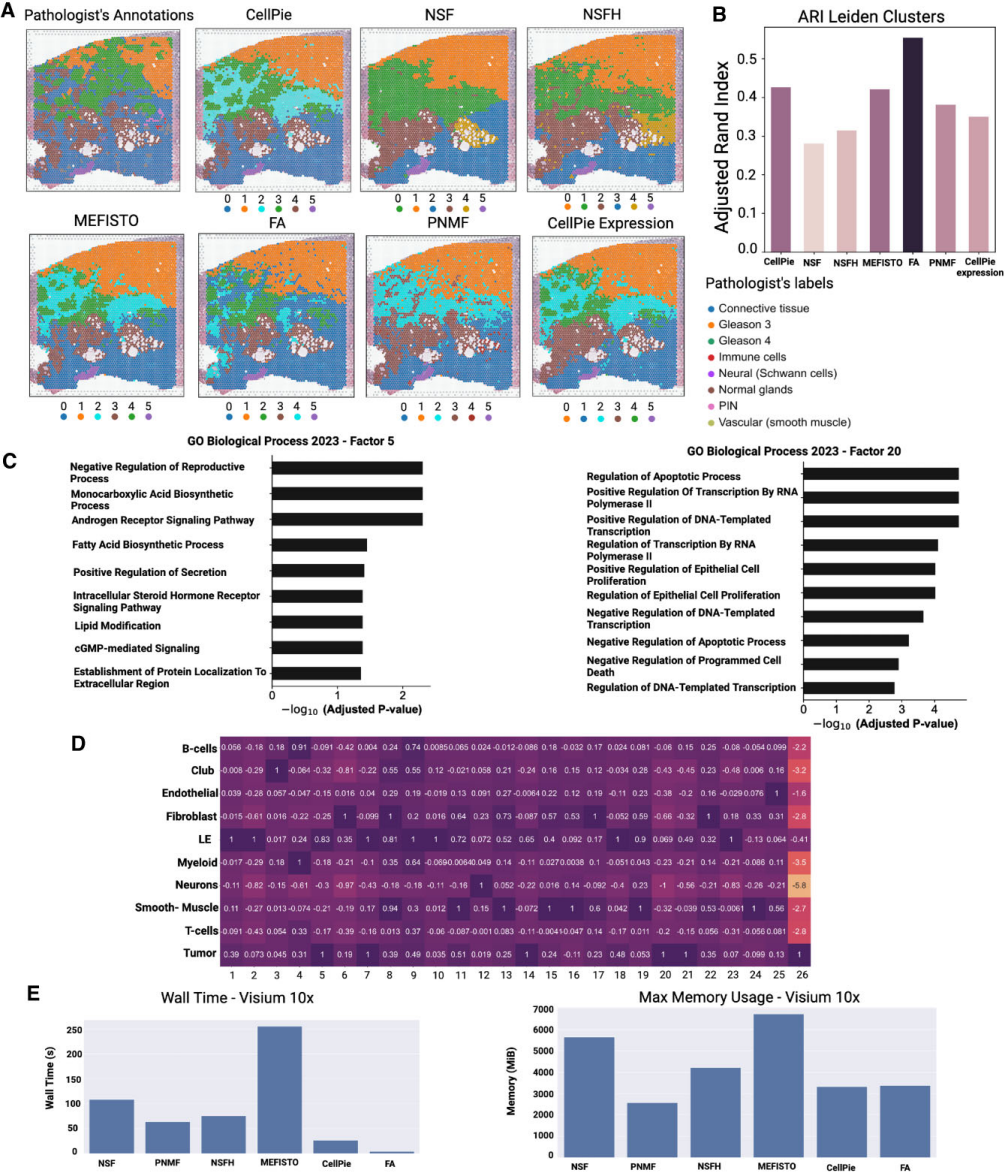
Comparison of *CellPie* against other published dimensionality reduction methods: (**A**) pathologist’s annotations and clustering results of *CellPie*, NSF, NSFH, MEFISTO, FA, PNMF,and *CellPie* with only gene expression. To cluster the factors of each method, the Leiden clustering algorithm with six clusters was used. (**B**) ARI between the pathologist’s annotations and the clusters of the methods. (**C**) Gene ontology of Factors 5 and 20, using *CellPie*’s top 150 marker genes associated with each of those factors. (**D**) Highest ranked cell types per factor, computed using a published single-cell RNA-seq dataset and Scanpy’s score$\_$genes function. (**E**) Running time (left) and maximum memory usage (right) for all the methods.

We then benchmarked full *CellPie*, NSF, NSFH, MEFISTO, FA, PNMF, and gene-only *CellPie* (corresponding to the standard single NMF), against the pathologist’s ground truth. It is worth mentioning here that among those methods, only *CellPie* provides the user with a model selection method. For fairness, we ran all the other methods for a range of factors (8–50). We were only able to run MEFISTO for 8–40 factors due to memory issues (kernel died). For each of the methods, we selected the number of factors that resulted in the maximum ARI between the factors’s Leiden clustering and the ground truth (Supplementary Fig. S2). Figures 3A and B illustrate the clustering results, where *CellPie* demonstrates the second highest ARI, just behind FA. However, *CellPie* excels in identifying the tumour region, particularly in distinguishing more Gleason 4 area at the Gleason 3 and Gleason 4 boundary, outperforming other methods. This finding is further supported by the contingency tables, where the highest score for both Gleason 3- and Gleason 4-associated clusters is found by *CellPie* (Supplementary Fig. S3 and Fig. 2G). Besides, FA’s high ARI is mostly because it assigns the whole connective tissue area into a single cluster, while the rest of the methods assign that area into two, or more, clusters. Therefore, when we focus on the consistency of the clustering results with the pathologist-annotated tumour regions, FA’s performance is less impressive. In contrast, *CellPie* not only maintains a high ARI but also demonstrates better Gleason 4 identification capability. This indicates that combining imaging and gene expression enhances the ability to identify tissue regions that are typically difficult to accurately recognize.

To further annotate the factors with cell types, we interrogated *CellPie*’s gene loading matrix in conjunction with a modified cell signature obtained from [27], with the addition of markers for B-cells [28] and neurons (using scanpy.tl.score$\_$genes function). In Fig. 3D, the factor interpretation of the invasive prostate carcinoma dataset is shown. Factor 5 exhibits the highest signal in tumour regions, encompassing both Gleason 3 and Gleason 4 areas, accompanied by high expression of *KLK2* and *KLK3* (Supplementary Fig. S4), which are markers of prostate glandular epithelium. This is consistent with Fig. 3D, where Factor 5 shows the highest correlation with both tumour and LE (luminal epithelial). Factor 10 displays the highest signal in normal glands, with high expression of *MSMB* and *AZGP1*, which are reported to be significantly downregulated in prostate cancer [29, 30]. This aligns with its lower tumour correlation and higher LE correlation in Fig. 3D. Tumour cells are mainly found in Factors 5, 7, 14, 20, and 21 (Fig. 3D).

To further validate the biological significance of the regions identified by our method at the molecular level, we applied Gene Ontology (GO) analysis (Fig. 3C). *CellPie* has demonstrated a particular advantage in the prostate cancer data by identifying a Gleason 4-specific factor, Factor 20. The main GO term enriched by this factor is associated with apoptotic processes and cell proliferation, such as ‘Positive Regulation of Epithelial Cell Proliferation’ and ‘Negative Regulation of Apoptotic Process’, indicating that the cancer cells at this stage have escaped programmed cell death and entered a rapid proliferation phase. This is consistent with the higher cancer progression observed in Gleason 4.

Factor 5 also exhibits tumour specificity. Unlike Factor 20, it covers regions, including both Gleason 3 and Gleason 4, while the normal connective tissue surrounded by the tumour shows either no signal or low signal. GO analysis reveals that the main enriched terms include ‘Negative Regulation of Reproductive Process’, ‘Androgen Receptor Signaling Pathway’, and ‘Monocarboxylic Acid Biosynthetic Process’. These terms are closely related to the progression of prostate tumours. The androgen receptor signaling pathway is a classic pathway that induces prostate cancer, and as prostate cancer progresses, significant changes occur in metabolic pathways, including glucose metabolism, lipid metabolism, and alterations in 1C metabolic homeostasis. These processes are highly related to the monocarboxylic acid biosynthetic process [31]. The negative regulation of the reproductive process is associated with the dedifferentiation of cells at this stage, leading to the loss of their original biological functions.

Analysis of the top genes within Factor 20 reveals that most of these genes have already been reported to be involved in the development of prostate cancer. For example, the genes *TRIB1*, *NR4A1*, and *NR4A2* are significantly upregulated in prostate cancer and can serve as biomarkers [32–34]. More importantly, some top genes, such as *GDF15*, are involved in the TGF-β signalling pathway, which has been reported to be involved in prostate cancer metastasis [35, 36], suggesting an increased invasive and metastatic capability of tumour cells at this stage. The *FOSB* gene, which also participates in the TGF-β signalling pathway, is required for migration and invasion in prostate cancer cells [37].

Factor 12 may be the Schwann cell-specific factor. Based on Fig. 2E and Fig. 3D, Factor 12 shows the best consistency with the regions corresponding to neurons. This is consistent with the enrichment analysis results. The GO terms enriched in Factor 12 are primarily related to nervous system development and myelination (Supplementary Fig. S5).

Analysis of the top genes in Factor 12 reveals some genes specifically expressed in the myelin sheath (Supplementary Fig. S5). For example, the calcium-binding protein *S100B* is a common marker for Schwann cells, and the *MPZ* gene is specifically expressed in Schwann cells, being a major structural protein in the myelin sheath [38].

Factor 13 exhibits high expression in normal connective tissue and has enriched GO terms that include ‘Homotypic Cell–Cell Adhesion’, ‘Supramolecular Fiber Organization’, and ‘Negative Regulation of Smooth Muscle Cell Proliferation’ (Supplementary Fig. S5). These terms highlight smooth muscle and collagen fibre intra- and extracellular-organization, which are key components of the connective tissue found in the prostate.

The signal of Factor 10 is primarily concentrated in normal glands, which is consistent with the enrichment of ion response in the GO terms. Zinc ions can be stored in prostate glands. Maintaining high concentrations of zinc ions is associated with normal prostate function, while the loss of zinc ion concentration can serve as a marker for prostate tumours [39]. Similarly, copper and calcium ion concentrations have also been reported to be associated with prostate tumours [40, 41]. These findings illustrate that the factors identified by *CellPie* can assist biologists in gaining deeper insights into the gene regulatory differences associated with the spatial architecture.

Running time and maximum memory usage across all the methods are shown in Fig. 3E. FA is the fastest method, followed by *CellPie*, while PNMF was found to be the most memory-efficient application, followed by *CellPie*.

### HER2-positive breast cancer

**Figure 4.**
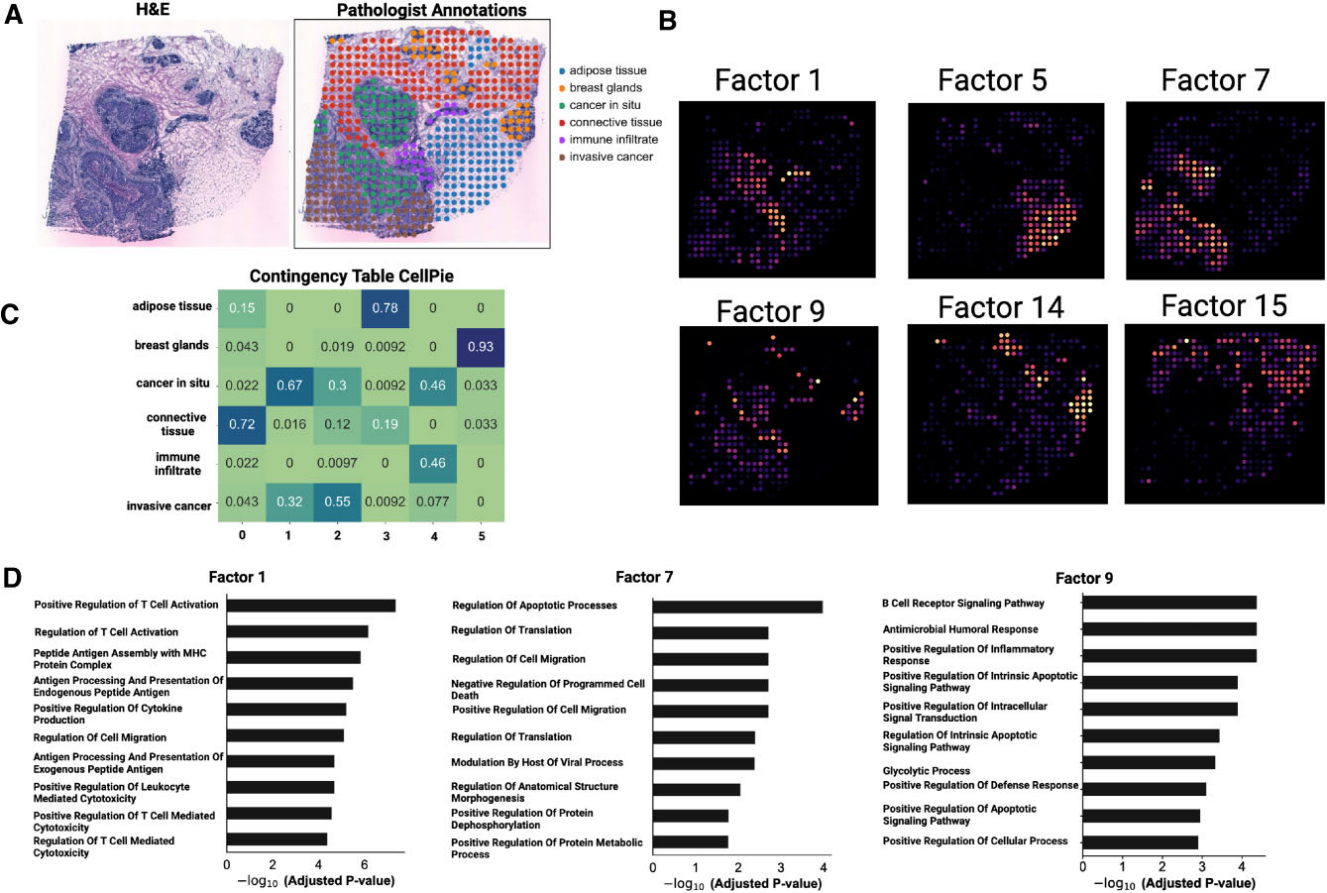
(**A**) H&E image of the HER2-positive breast cancer sample (patient H1) and pathologist’s annotations. (**B**) *CellPie*’s selected factors. (**C**) Contingency matrix between *CellPie*’s factors and pathologist’s annotations. (**D**) Gene ontology of Factors 1, 7, and 9.

For the second validation, we used a published ST HER2-positive breast cancer dataset (patient H1) [22], where pathologist annotations are available and were used as ground truth (Fig. 4A). The regions identified by the pathologist’s were labelled as adipose tissue, breast glands, cancer *in situ*, connective tissue, immune infiltrate, and invasive cancer. To select the optimal number of factors, we used *CellPie*’s BCV model selection method, where the optimal number was found to be *k* = 16 (Supplementary Fig. S6A), while the optimal modality weight was found to be *α* = 0.3. With these settings, the resulting factors are shown in Fig. 4B and Supplementary Fig. S7.

To benchmark against the other methods, we followed a similar strategy as in the prostate cancer case, where we ran all the methods across a range of number of factors and selected the number that led to the optimal ARI (Supplementary Fig. S8).

**Figure 5.**
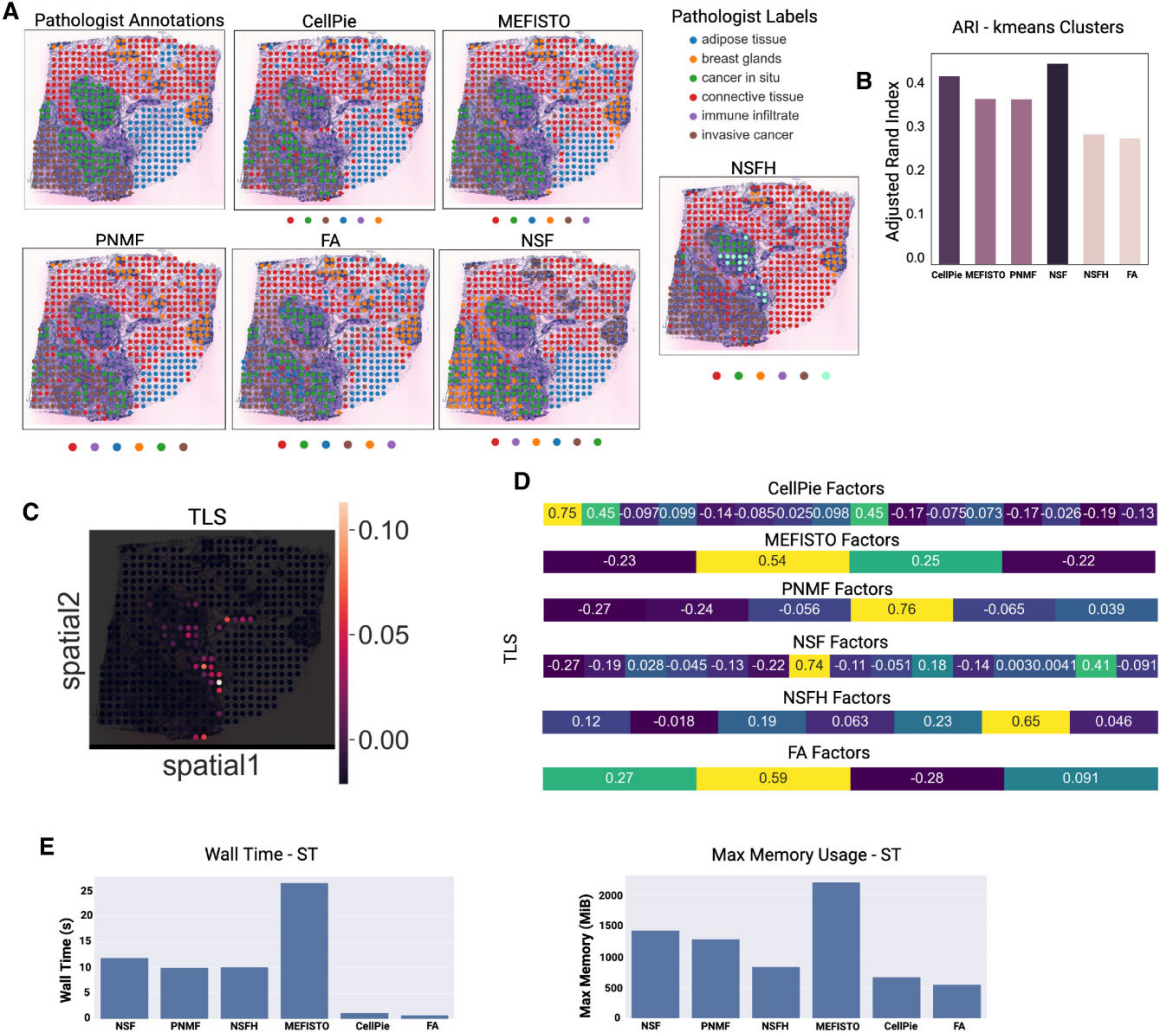
(**A**) Pathologist’s annotations and clustering results of *CellPie*, MEFISTO, PNMF, FA, NSF, and NSFH factors. To cluster the factors of each method, the *k*-means clustering algorithm with six clusters was used. (**B**) ARI between the resulting clusters and the ground truth. (**C**) Spatial distribution of the tertiary lymphoid structure (TLS) score, computed using the results in [22]. (**D**) Pearson correlation between *CellPie*’s, MEFISTO’s, PNMF’s, NSF’s, NSFH’s, and FA’s factors and the TLS scores. (**E**) Running time (left) and maximum memory usage (right) for all the methods.

To compare the resulting factors of each method with the ground truth pathologist’s annotations, we clustered the factors using *k*-means algorithm with *k* = 6 clusters, so that it reflects the number of the ground truth labels (Fig. 5A). The top two-performing methods are NSF (ARI 0.46) followed by *CellPie* (ARI 0.43), while the rest of the methods show lower ARI (Fig. 5B). Contingency tables show that most of the methods distinguish breast glands, immune infiltrates, adipose tissue, and connective tissue as mainly being single clusters, while cancer *in situ* and invasive cancer regions are a mix of two or more clusters (Fig. 4C and Supplementary Fig. S9).

It has been shown that in HER2-positive tumours, TLS have predicted values for clinical outcomes and treatment responses. These structures, which resemble lymph nodes, can form ectopically in tissues such as tumours, providing anti-tumour immune responses [22, 42]. As shown in [22], TLS sites are enriched in B and T cells. We used the TLS score results provided in [22] to investigate whether any of the resulting factors aligns with the TLS-enriched areas (Fig. 5C). For each method, we computed the Pearson correlation between the TLS score and each of the factors (Fig. 5D), and we found that the largest correlation (0.76) was in PNMF’s Factor 4, while *CellPie* shows the second highest correlation (0.75) for Factor 1 (corresponds to the immune infiltrate region).

The enrichment results of Factor 1 mainly focus on items related to T-cell-mediated immune responses. From Fig. 4B, the areas corresponding to Factor 1 include immune infiltrates, cancer *in situ*, with the strongest signal observed in the immune infiltrates. This is consistent with previous reports that breast cancer tissues contain infiltrates dominated by activated T lymphocytes [43]. This finding is consistent with one of the most enriched terms in the GO analysis, the ‘Positive Regulation of T Cell Activation’ (Fig. 4D). Besides, the region covered by Factor 9 includes immune infiltrates and parts of the breast area. The enriched terms obtained are mainly focused on B-cell immunity (Fig. 4D). The overlapping region aligns with the structural characteristic of TLS, where T cells surround B cells (Fig. 4B and Fig. 5C).

Factor 7 overlaps highly with two cancer areas, cancer *in situ* and invasive cancer. The enriched GO terms include cell migration, apoptosis, and protein metabolic processes, which are consistent with the increased infiltration capability of cancer cells at this stage (Fig. 4D).

In Fig. 5E, we show running time and maximum memory usage for all the methods. Even though for this type of datasets, all the methods require a small amount of time and memory to run, *CellPie* and FA are the two fastest methods (with a few seconds difference) as well as the two methods that require the least amount of memory for their application. However, as we will see in the next application, for very highly resolved datasets, most of these methods become inapplicable.

### Visium HD human colorectal cancer

Visium HD provides single-cell or near-single-cell spatial resolution, depending on the bin size, offering a comprehensive view of spatial gene expression patterns. However, due to the high dimensionality of the data (resolution at 2, 8, and 16 μm), most of the current factor analysis methods require either a very long time to run on a standard computer or become impractical.

**Figure 6.**
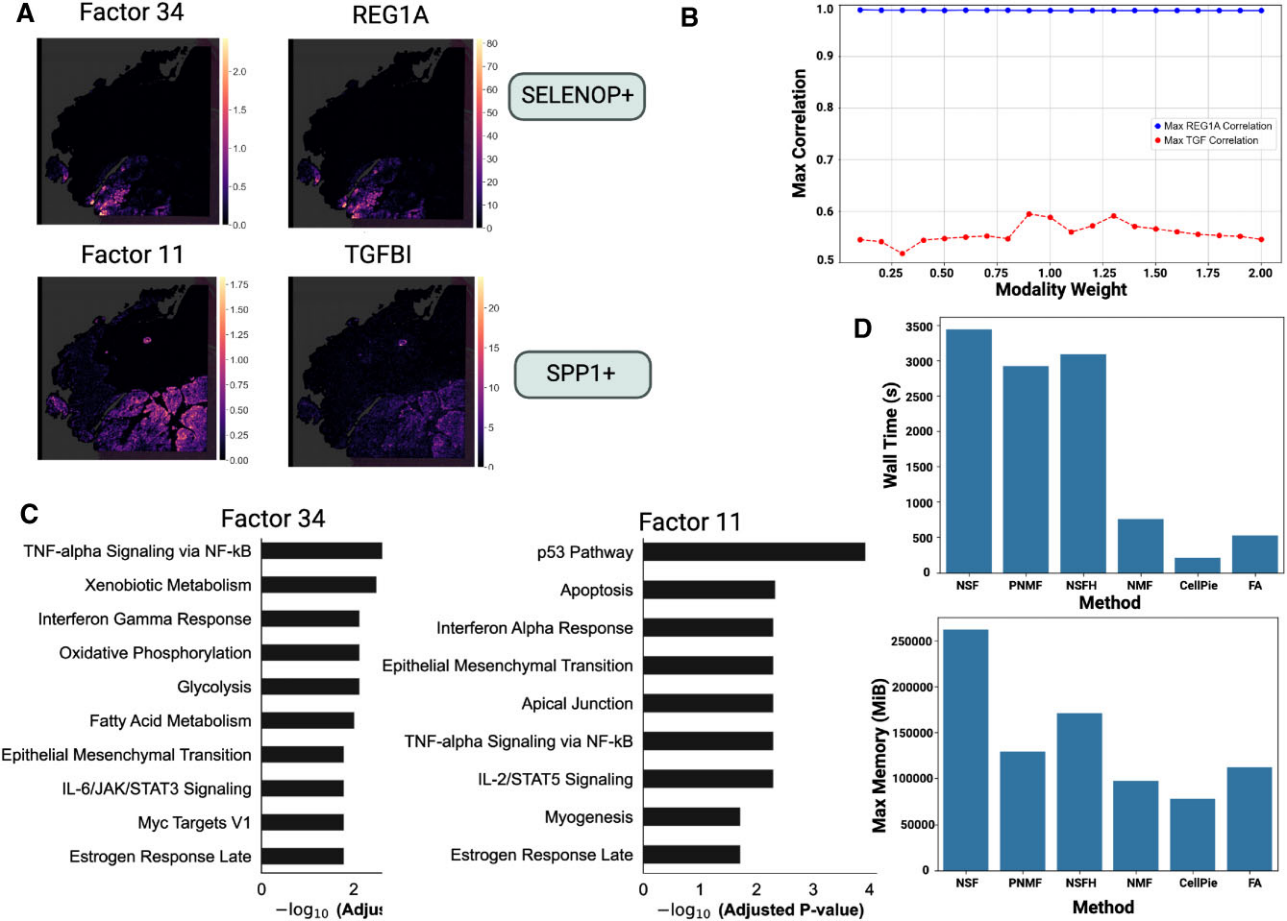
(**A**) Top: *CellPie*’s Factor 34 and spatial gene expression of *REG1A* gene, marker of SELENOP+ macrophages. Bottom: *CellPie*’s Factor 11 and spatial gene expression of *TGFBI* gene, a marker of SPP1+ macrophages. (**B**) Pearson correlation between *CellPie*’s Factors 34 and 11 and the *REG1A* and *TGFBI* genes, respectively, across a range of weights. (**C**) Pathways enriched in Factors 34 and 11, respectively, where the MSigDB Hallmark 2020 database was used. (**D**) Running time (top) and maximum memory usage (bottom) for NSF, NSFH, PNMF, *CellPie*, FA, and sklearn-NMF.

We applied *CellPie* to a Visium HD human colorectal cancer (CRC) dataset from patient P1 [9]. Given the key role of the immune cellular landscape in the CRC progression, in their paper, the authors studied the immune cellular composition of the tumour microenvironment of the CRC. Among other immune cellular populations, macrophages were found to be the most abundant in the tumour periphery. In that region, the authors identified two pro-tumour macrophage subpopulations forming distinct spatial niches. These populations are mainly defined by the expression of *SELENOP* and *SPP1* genes, therefore the authors defined the two distinct macrophage subpopulations as SELENOP+ and SPP1+. Differential gene expression showed enrichment of the *REG1A* and *TGFBI* genes for the SELENOP+ and SPP1+, respectively. Therefore, we wanted to test whether *CellPie* can identify factors that are related to these two macrophage types. We ran *CellPie* on the 16 μm bin resolution for 80 factors across a range of different modality weights and computed the Pearson correlation between the resulting factors and the expression of *REG1A* and *TGFBI* genes (Fig. 6B). For all the tested weights, there is a very high correlation between Factor 34 and REG1A expression (0.99), while the correlation between SPP1+-associated factor (Factor 11) and *TGFBI* was lower (maximum 0.59) (Fig. 6A and B). The correlation for *REG1A* ranges from 0.98 to 0.99 across the weights; however, for *TGFBI* we found that the correlation ranges from 0.54 to 0.59, where the lower corresponds to *CellPie* with no image features (*α* = 2.0), while the highest corresponds to modality weight *α* = 0.9, highlighting the importance of integrating image and gene expression features.

Unlike enrichment analysis using differentially expressed genes, we identified factor-specific terms using the top genes within each topic to demonstrate whether *CellPie* can be used to analyse the tumour microenvironment. As shown in Fig. 6C, besides the common tumour-related terms such as TNF-α signalling via NF-κB and epithelial–mesenchymal transition, a major difference between Factor 34 and Factor 11 lies in the IFN (interferon) system. Factor 34 is enriched for terms related to type-2 IFNs (IFN-II, including interferon gamma), whereas Factor 11 is enriched for terms related to type-1 IFNs (IFN-I, including interferon alpha). These two types of IFNs differ in their sources and functions, and their effects are concentration-dependent. For instance, tumour cells can produce low concentrations of IFN-I to avoid DNA damage and promote tumorigenesis, while low concentrations of IFN-II may enhance metastasis, consistent with the function of REG family genes [44–46]. This result not only reveals differences in the tumour microenvironment between the two regions but also suggests that different regions of the tumour may require distinct therapeutic strategies and target selections.

We also investigated the running time of NSF, NSFH, PNMF, *CellPie*, FA, and sklearn-NMF. Most of the methods failed to run on a standard MacBook laptop, so we used HPC settings with 16 cores and 512 GB memory. MEFISTO failed to run under these conditions, so we removed it from the benchmark. For high-resolution datasets, such as Visium HD, the optimal number of factors is usually large (based on BCV for *CellPie*, it is around 100) (Supplementary Fig. S6C). NSF and NSFH could not be run with these many factors due to memory and runtime constraints. To facilitate a comparison of the model’s runtime and memory usage, we ran all models with 30 factors. However, in a practical setting, using fewer factors would likely produce a worse factorization. In addition, NSF, NSFH, and MEFISTO are based on inducing points, and hence, the quality of the results is related to the number of inducing points used. Unfortunately, a high number of inducing points, required for very highly resolved datasets, made these methods inapplicable due to runtime and memory constraints, and therefore we had to select a lower number of inducing points—3000 for both NSF and NSFH. For fairness, we ran all the methods using all the genes in the dataset and for 30 factors. For the 16 μm resolution, the results are shown in Fig. 6D. *CellPie* is the most efficient method for both running time and maximum memory usage. Using the same settings, we examined how those methods perform on the 8 μm resolution. At this resolution, only *CellPie*, sklearn-NMF, and FA could run (Supplementary Fig. S10). *CellPie* was the most efficient method at this resolution in both maximum memory usage and runtime. These results highlight the superior scalability of *CellPie* against the other methods.

## Discussion

The field of spatial omics moves towards producing highly resolved multimodal datasets. In this paper, we presented *CellPie*, a scalable and efficient multimodal factor discovery method for ST that leverages a fast implementation of NMF that integrates ST gene expression data with histopathological image features. For high dimensional datasets, we showed that *CellPie* significantly reduces the computational time and memory requirements in comparison to other methods, making it applicable to the latest highly resolved ST datasets, such as those from the Visium HD technology.

Our evaluation on two different human cancer types and spatial resolutions demonstrated that *CellPie* is among the top-performing methods, achieving performance close to the best method in terms of the overall clustering accuracy. Furthermore, *CellPie* outperforms existing methods in the identification of Gleason 3 and Gleason 4 areas in the invasive prostate carcinoma dataset. In the HER2-positive breast cancer dataset, *CellPie* effectively identified key regions corresponding to different tissue types and showed the second highest factor correlation to the TLS structures. Furthermore, in the Visium HD CRC dataset, *CellPie* identified factors associated with distinct macrophage populations with specific gene expression patterns while maintaining superior computational efficiency.

The integration of histopathological features with gene expression has been shown to improve the accuracy of the ST factor analysis. By adjusting the relative weight of the two modalities, *CellPie* provides a flexible approach to factor discovery, enabling the extraction of meaningful spatial patterns from complex tissue samples.

One limitation is that *CellPie* does not explicitly model the spatial dimension of the data, such as the neighbourhood relationships between the spots. Hence, a spatially aware version of the *CellPie*would be an interesting future direction. Furthermore, *CellPie* and the intNMF algorithm [18], which *CellPie* is based on, could be extended to jointly factorize more than two matrices simultaneously. This enhancement would allow for integration of several omics datasets possessing a shared dimension (for example including spatial protein markers).

## Supplementary Material

gkaf251_Supplemental_File

## Data Availability

The *CellPie* code and the notebooks to reproduce the analysis are available as an open-source Python package at https://github.com/ManchesterBioinference/CellPie and https://doi.org/10.6084/m9.figshare.28570457. The prostate invasive carcinoma pathologist’s annotations are also available on the GitHub repository. The human prostate cancer can be downloaded from https://www.10xgenomics.com/datasets/human-prostate-cancer-adenocarcinoma-with-invasive-carcinoma-ffpe-1-standard-1-3-0, the HER2-positive can be found in https://github.com/almaan/her2st, while the Visium HD colorectal cancer can be downloaded from https://www.10xgenomics.com/products/visium-hd-spatial-gene-expression/dataset-human-crc.
