# Supplementary material for: CellPie: a scalable spatial transcriptomics factor discovery method via joint non-negative matrix factorization
Source: Nucleic Acids Res. 2025 Apr 1;53(6):gkaf251. doi: 10.1093/nar/gkaf251 (PMC12086691; doi:10.1093/nar/gkaf251)
Supplement: gkaf251_Supplemental_File [file gkaf251_supplemental_file.pdf]

Supplementary material for '*CellPie*: a scalable spatial transcriptomics factor discovery method via joint non-negative matrix factorization '.

Sokratia Georgaka[1,*], William Geraint Morgans[1], Qian Zhao[1], Diego Sanchez Martinez[2], Amin Ali[1,3], Mohamed Ghafoor[1], Syed-Murtuza Baker[1], Robert Bristow[2,4], Mudassar Iqbal[1] and Magnus Rattray[1,*]

[1]Division of Informatics, Imaging and Data Sciences, Faculty of Biology, Medicine and Health, University of Manchester, Manchester M13 9PL, UK
[2]Cancer Research UK Manchester Institute, Manchester
[3]The Christie NHS Foundation Trust, Manchester
[4]Division of Cancer Sciences,Faculty of Biology, Medicine and Health, University of Manchester, Manchester M13 9PL, UK

March 10, 2025

1 Parameter settings

For the spatial transcriptomics data, we used normalised and logarithmised counts for *CellPie*, using Scanpy's normalize_total and log1p functions. The same normalisation was used for *MEFISTO*, *FA* and *NMF*. For the image features we used sklearn's MinMaxScaler which transforms features by scaling each feature to be between zero and one.. We note that for the Visium HD datasets we used raw gene expression counts. We used raw counts for *NSF*,*NSFH* and *PNMF*. As in *NSF* paper, we used the top $2,000$ informative genes using Poisson deviance for *NSF*,*NSFH*,*PNMF* and *MEFISTO*. We ran *NSF* and *NSFH* using $3,000$ inducing points with Poisson likelihood for prostate cancer and crc datasets and 500 inducing points for the HER2-positive breast cancer dataset. For *MEFISTO* we used Gaussian likelihood with $1,000$ inducing points for the Visium and Visium HD datasets and 500 for the ST dataset. Scripts to reproduce the results are available in `https://github.com/ManchesterBioinference/CellPie`. For computational time comparison purposes only, we ran *NSF*, *NSFH*, *PNMF*, *FA*, *NMF* and *CellPie* on the Visium HD CRC dataset with 30 factors, using raw counts. For the *NSF* and *NSFH*, as the 'preprocess.deviancePoisson' failed for that dataset, we used all the genes in the dataset and we filter only those which are expressed in at least one spot and with at least 100 counts across all the spots.

2 Supplementary Figures

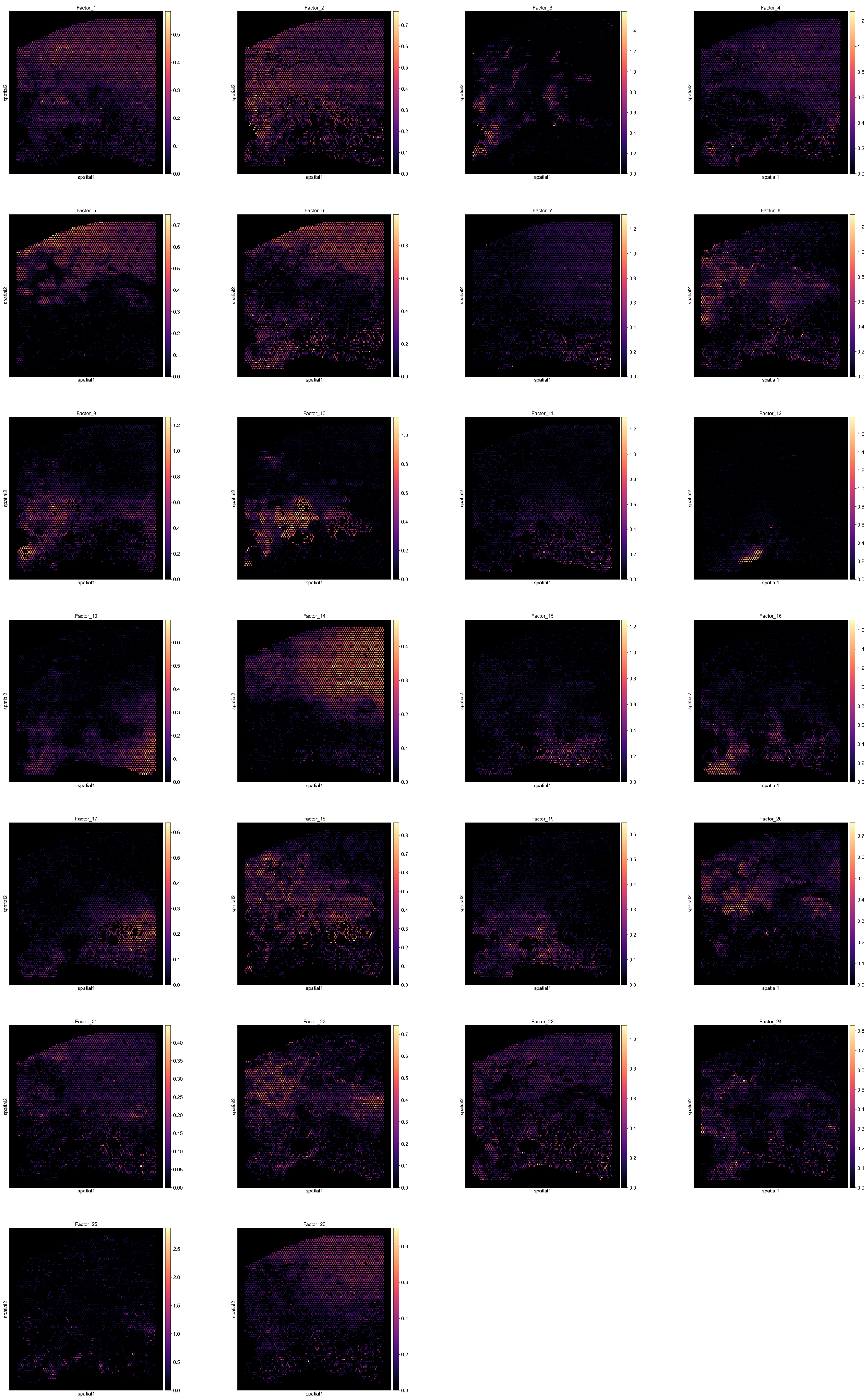

Figure 1: CellPie Factors for the human prostate adenocarcinoma with invasive carcinoma dataset.

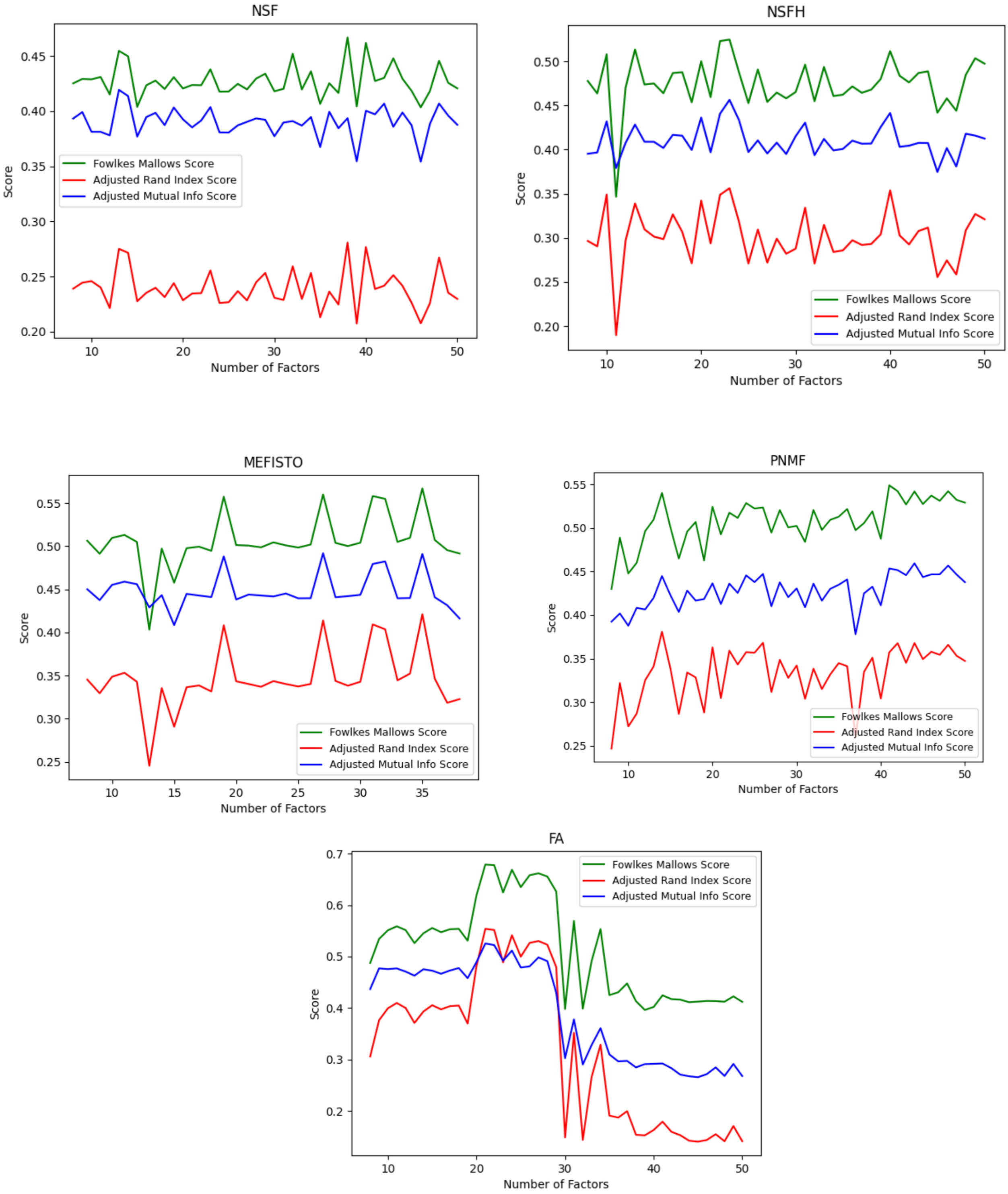

Figure 2: Model selection for the human prostate adenocarcinoma with invasive carcinoma dataset for NSF, NSFH, FA, MEFISTO and PNMF. Each method was run for a range of factor number, between $8-50$ - MEFISTO between $8-40$.

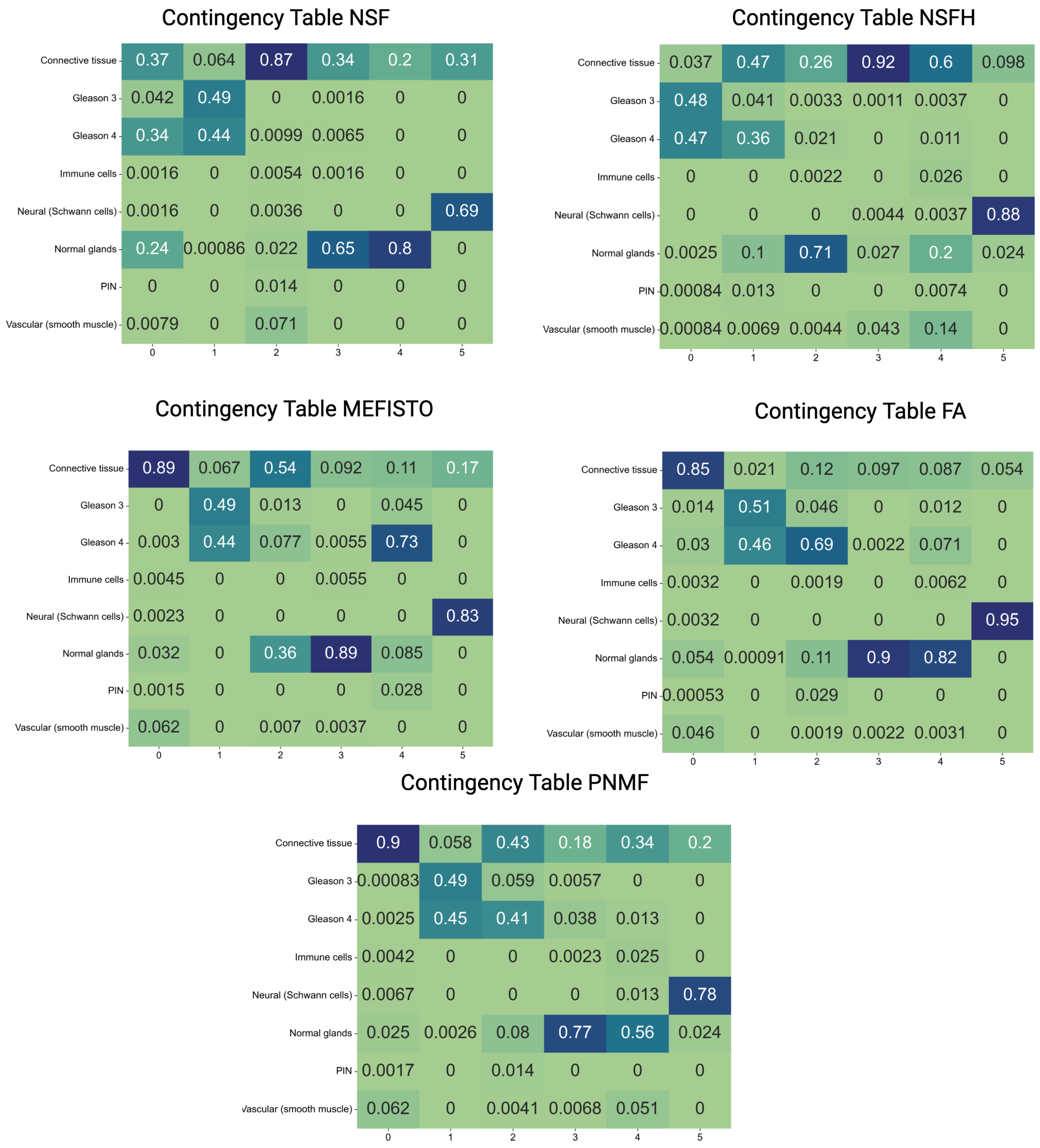

Figure 3: Contingency tables for NSF, NSFH, FA, MEFISTO and PNMF for the human prostate adenocarcinoma with invasive carcinoma dataset.

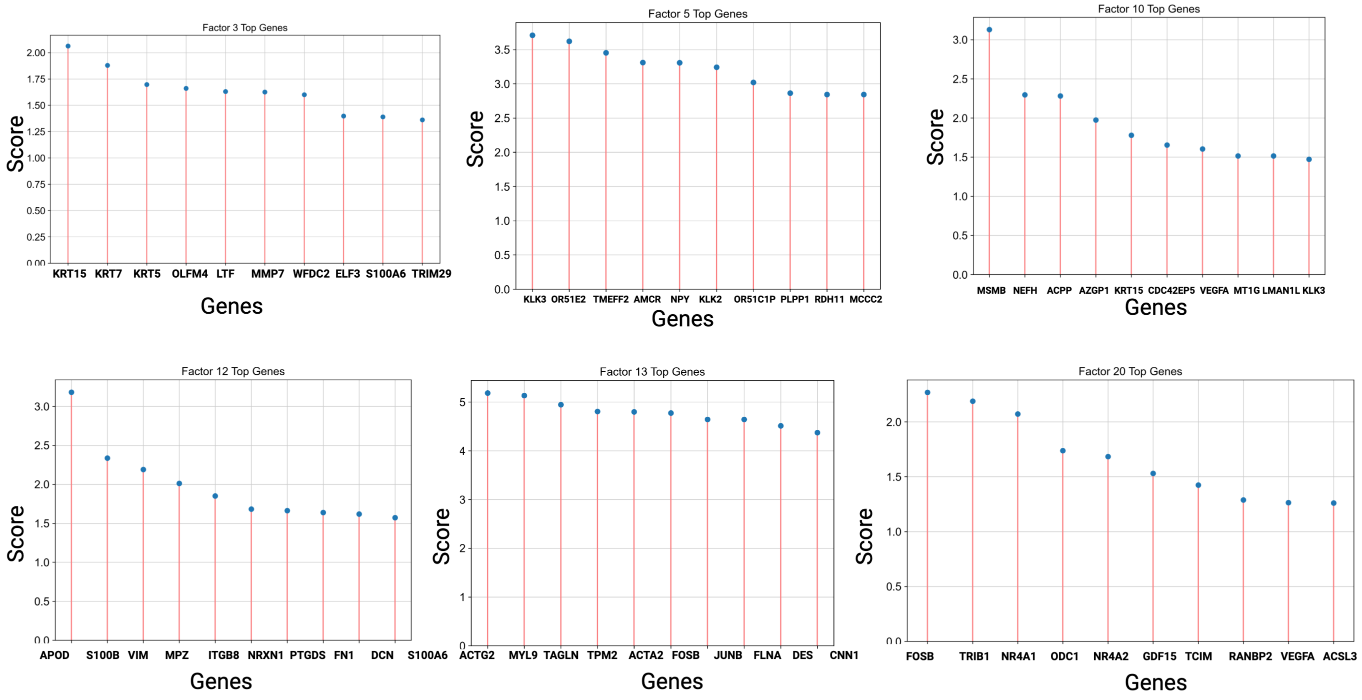

Figure 4: Plots showing the score of the top 10 marker genes of Factors 3,5,10,12,13 and 20.

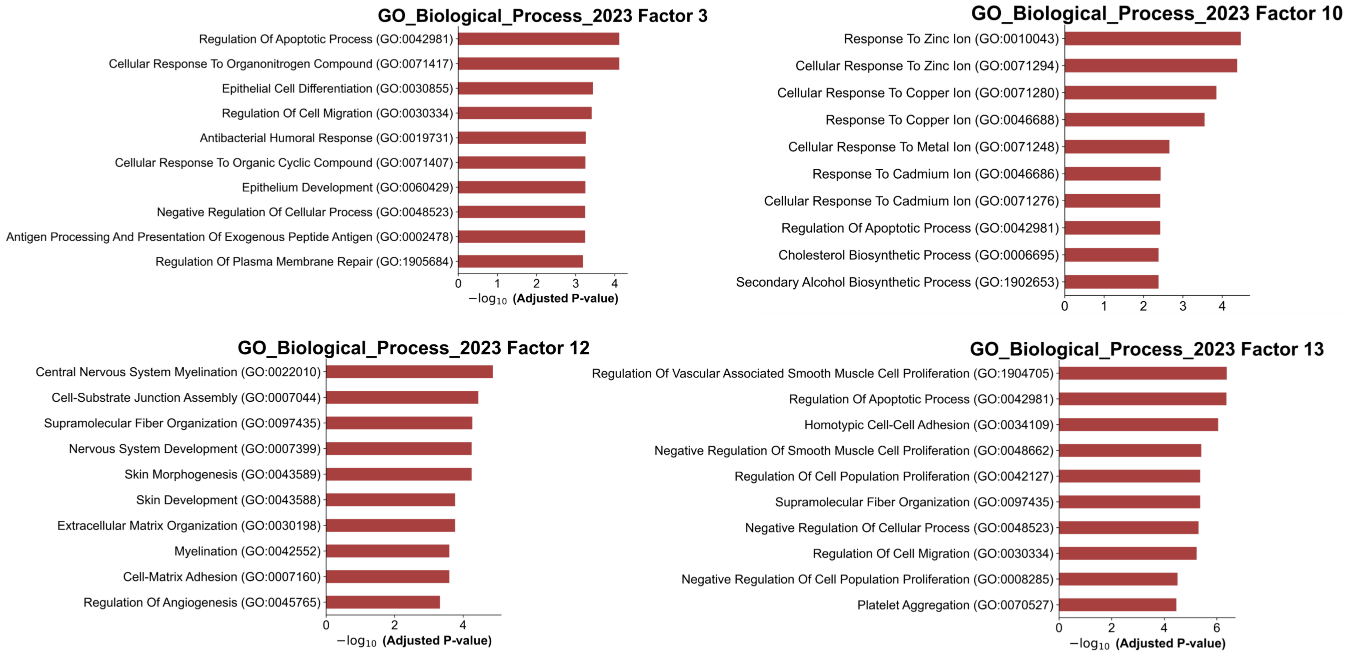

Figure 5: Gene ontology (Biological Processes 2023) for Factor 3, Factor 10, Factor 12 and Factor 13 of the human prostate adenocarcinoma with invasive carcinoma dataset.

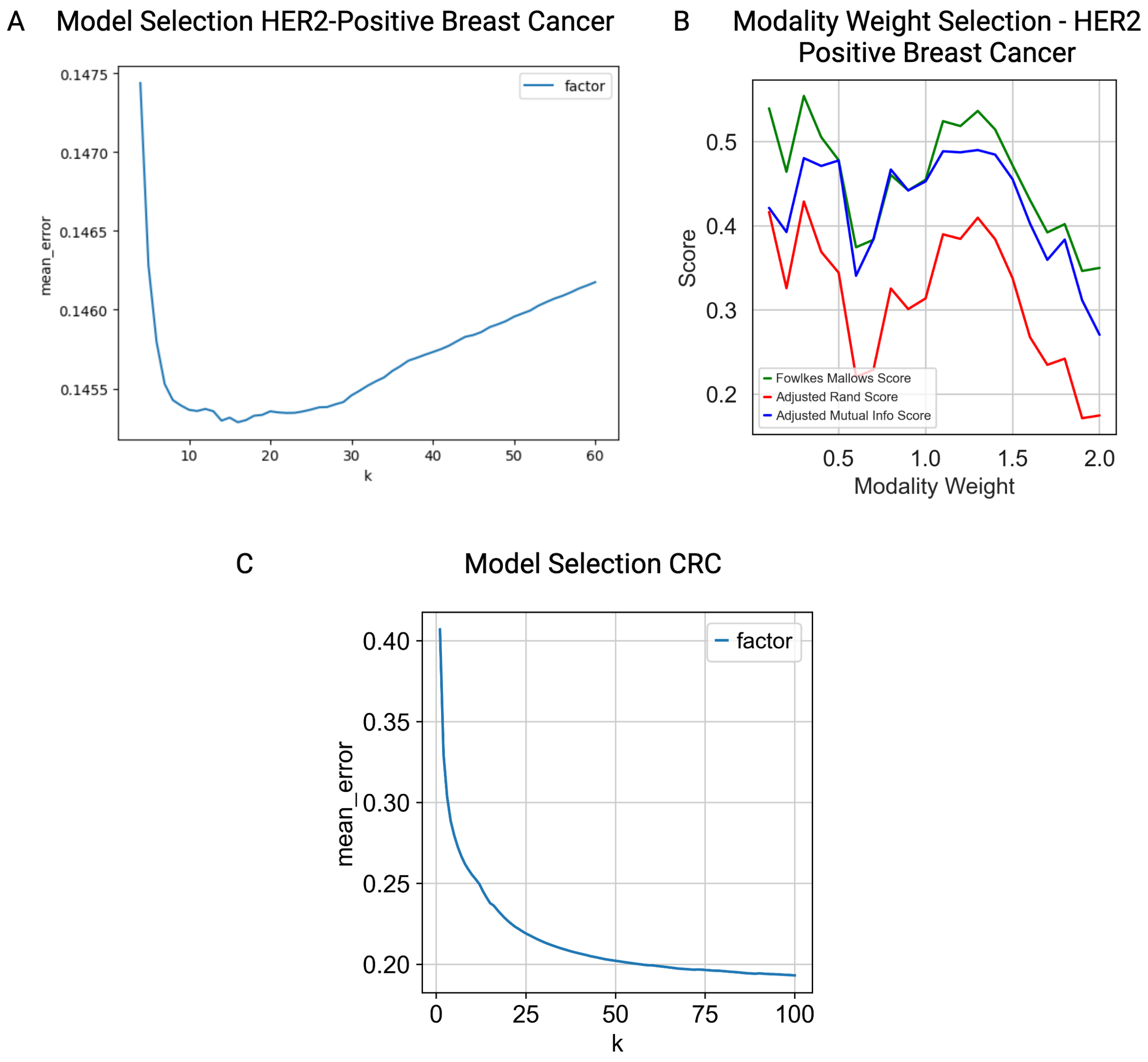

Figure 6: (A) Model selection for the HER2-positive breast cancer dataset. (B) Modality weight selection for human HER2-positive breast cancer.(C) Model selection for the human colorectal cancer Visium HD dataset.

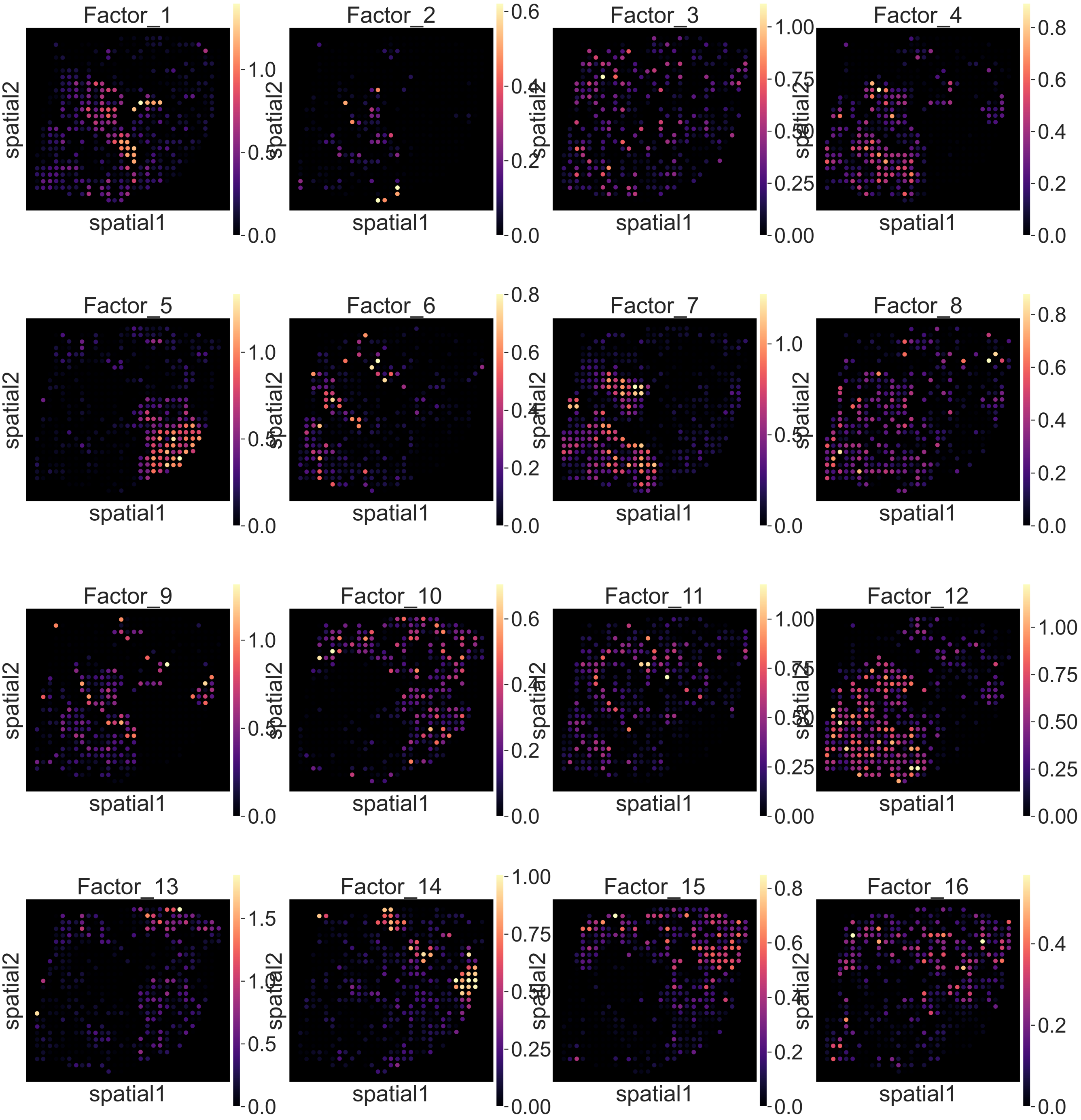

Figure 7: CellPie Factors for the HER2-Positive Breast Cancer dataset.

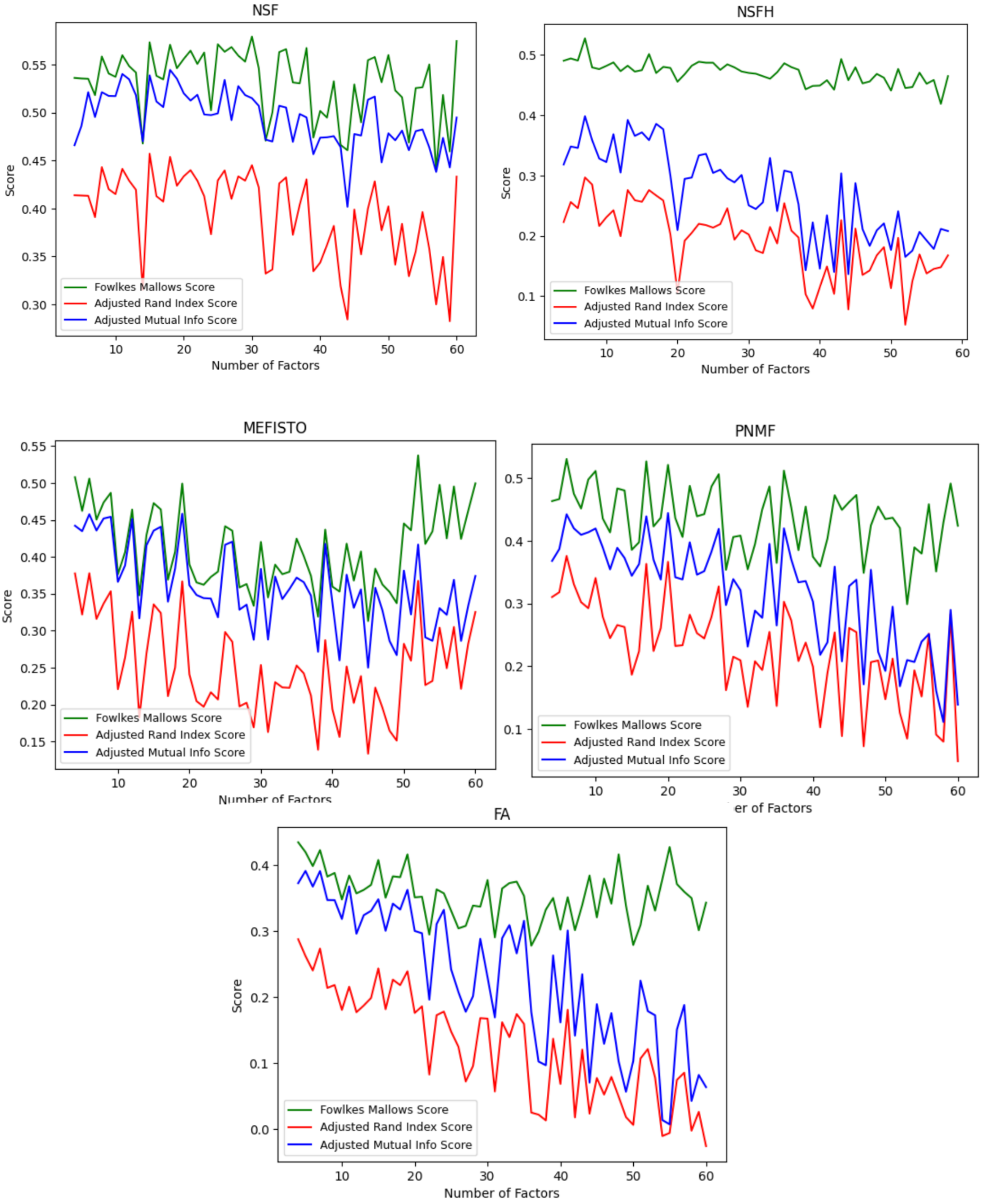

Figure 8: Model selection for the HER2-positive breast cancer dataset for NSF, NSFH, FA, MEFISTO and PNMF. Each method was run for a range of factor number, between $3 - 60$.

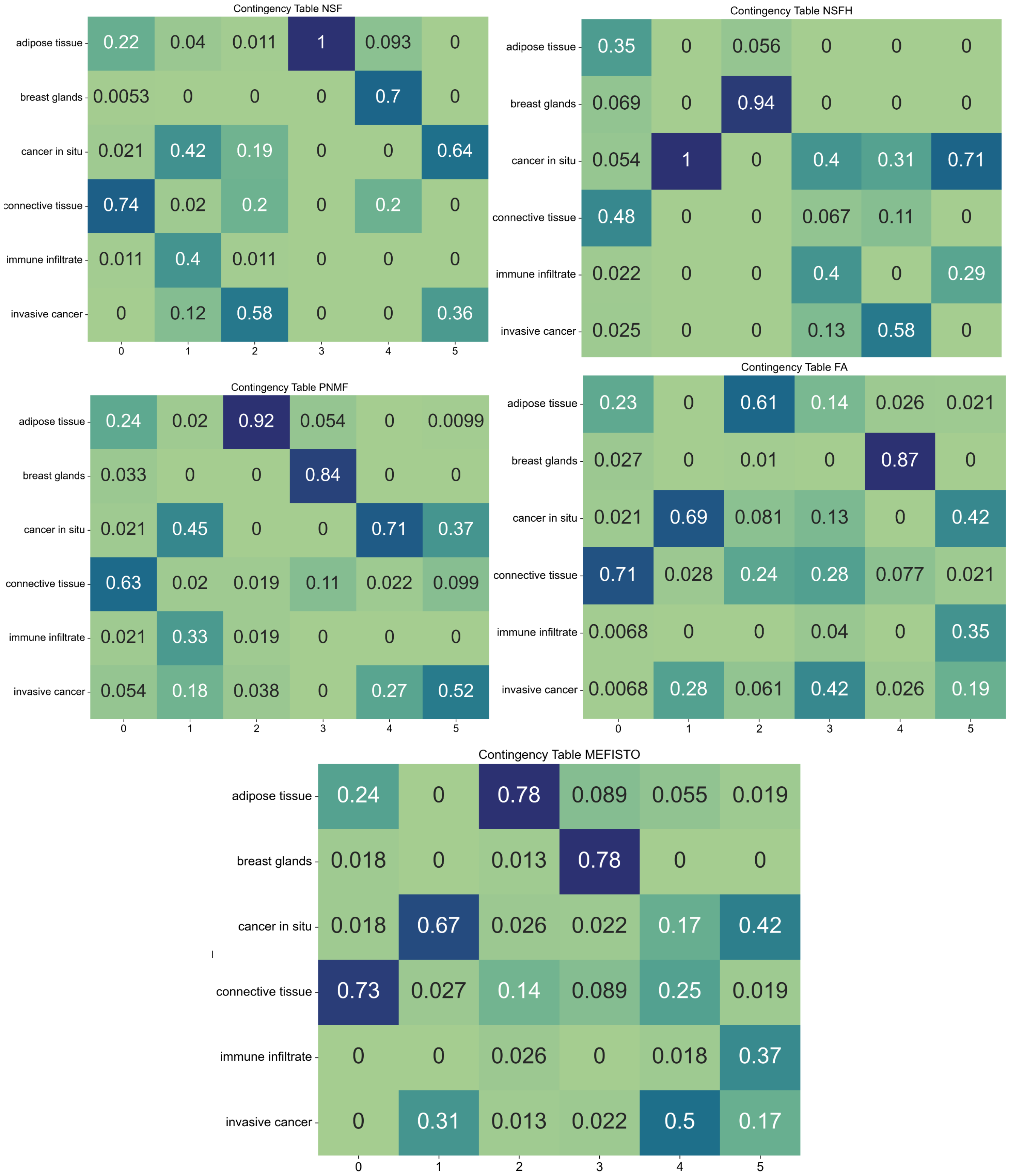

Figure 9: Contingency tables for NSF, NSFH, FA, MEFISTO and PNMF for the HER2-positive breast cancer dataset.

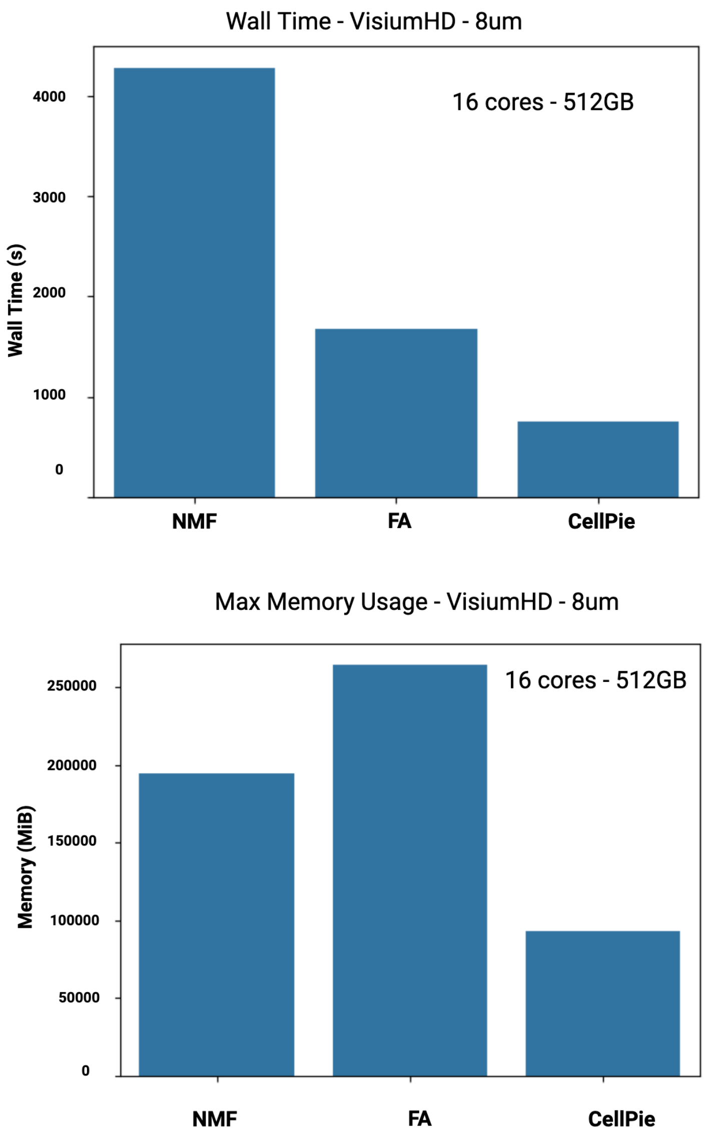

Figure 10: Running time and maximum memory usage for CellPie, FA nd NMF for the Visium 10x crc dataset with 8um resolution.
